# The challenge of describing the epidemiology of HTLV in the Amazon region of Brazil

**DOI:** 10.1186/s12977-020-0512-z

**Published:** 2020-02-14

**Authors:** Ricardo Ishak, Marluísa de Oliveira Guimarães Ishak, Antonio Carlos R. Vallinoto

**Affiliations:** Laboratório de Virologia, Instituto de Ciências Biológicas, Universidade Federal do Pará, Rua Augusto Corrêa no.1, Belém, Pará 66075-110 Brazil

**Keywords:** HTLV-1/2, Prevalence, Epidemiology, Amazon region, Brazil

## Abstract

HTLV-1 was the first described human retrovirus and was soon found to be associated with severe clinical diseases, including a devastating lymphoma/leukemia and other inflammatory diseases. Although HTLV-2 is not usually pathogenic, it is widely distributed among native Indian populations in Brazil, particularly in the Amazon region of the country. Presently, HTLV spreads mainly by the sexual route and from mother to child, and virus persistence is an active biological factor aiding its transmission. Recently, the use of illicit drugs has been shown to be an additional risk factor, showing the influence of new habits on the epidemiology of HTLV in the region. Despite the detection of the virus in several different populations in the Amazon region of Brazil for almost 30 years, the exact prevalence of HTLV-1/2 is not well defined. The original biases in sampling and the selection of epidemiologically unsuitable populations were commonly repeated in most prevalence studies, generating unreliable and conflicting figures that do not represent the actual prevalence of HTLV. The improvements in clinical and laboratory facilities have resulted in the description of several clinical manifestations that were previously unknown in the region. The extent of the spread of the virus must be defined in this region, which is the largest geographical area of the country. As prophylaxis advances toward the use of vaccines against HTLV-1, it is important to determine who is at risk of being infected and developing a disease to successfully implement preventive measures, particularly as proposals are made to eradicate the virus among humans.

## Background

Human T cell lymphotropic viruses 1 and 2 (HTLV-1 and HTLV-2) are medium-sized virus particles (80–120 nm) belonging to the family *Retroviridae*, genus *Deltaretrovirus* [1]. Presently, there are six molecular subtypes (namely, a, b, c, d, e and f) of HTLV-1 [2–4] and four (a, b, c and d) of HTLV-2 [5–8]. Two other types, HTLV-3 and HTLV-4, have been described [9] as examples of cross-species transmission in a geographically isolated forest area in Cameroon, but so far, neither have been detected elsewhere or have spread further [10, 11].

Retroviruses share similar biological and replicative properties, including the evolutionary aspect of viral and cell nucleic acid integration, viral persistence, viral latency and vertical transmission to the offspring. HTLV integrates the transcribed RNA as a DNA provirus into the cell nucleic acid [12–16], and this simple evolutionary procedure leads to the persistence of the virus and its maintenance in nature and has serious implications for the different clinical and epidemiological outcomes of the infection and diseases associated with the virus. The wide array of clinical outcomes shows the target complexity within the human host (including the CNS, blood, lungs, eyes, muscles, bladder and skin), and several medical specialties have to be involved in the care and treatment of infected and diseased persons [17–27].

HTLV is an ancient infection in humans and alternates between persistence and productive cycles, which favors an effective mechanism involving vertical and horizontal transmission. According to the geographical environment and behavioral risk factors, the increased risk of transmission of the virus increases the prevalence and incidence of infection and disease [6, 28–32].

Viral dispersion in the human body leads to the infection of several biological fluids, including the blood, semen, vaginal fluid, and milk and results in its vertical transmission from mother to child (via the placenta and perinatal breast feeding), the injection of drugs, the transfusion of blood and its components, the transplantation of organs and the engagement in sexual relations [29, 33–38]. Each of these plays an important role in viral maintenance. The risk associated with transfusion used to be major but it decreased sharply with the introduction of strict regulatory policies regarding blood screening in Brazil and elsewhere [39–41]. The recommended policy of avoiding breastfeeding in mothers who carry the virus is generally followed in urban areas and is an efficient procedure whereby transmission is reduced.

Vertical transmission is common and is probably the most important route for the maintenance of HTLV within epidemiologically closed communities, as seen with HTLV-2c among Indians communities in the Amazon area of Brazil and in urban areas [6, 33, 42–44]. Among the Guaymi in Panama, there are relatively more infected children born from infected mothers [28, 45], and among the Kubenkokre, Kayapo villagers in the Amazon region of Brazil, familial cluster studies have showed the transmission of the virus crossing one or more generations, and more than 20% of the children under 9 years of age are infected [6]. Molecular evidence has clearly showed that the virus is transmitted from mother to child among isolated Indian tribes, which illustrates the importance of the mechanism for the maintenance of the high endemicity of the virus [33, 36].

The geographical distribution of the virus is influenced by the transmission route used. In North America, HTLV-2 was probably spread from American Indians to injection drug users (IDUs), which resulted in the transmission of the virus to other IDU communities in Europe and was the most likely route that carried HTLV to Vietnam, during the war in 1960–1970 [46–49]. In the Amazon region of Brazil, injecting drugs was not an important route for the spread and maintenance of HTLV-1/2 in urban, nonurban, or isolated communities or in co-infection with HIV-1 [50, 51] in a clear contrast to what was usually seen in other areas of Brazil, where the use of illicit drugs is a well-known risk factor for both virus infections [52–54]. More recently, high prevalence rates and levels of genetic diversity of both HTLV-1 and HTLV-2 were shown among illicit drug users in the State of Para [55], which is a change in the epidemiological pattern of the spread of the two viruses in the region.

Sexual transmission is certainly the most important transmission route for HTLV-1 and HTLV-2 and serves as an efficient mechanism for the spread of HTLV-2c among native Indian groups [6, 28, 56]. In urban areas, HTLV infection is more common among women [42, 57, 58]. Within epidemiologically closed communities such as Indian populations, the distribution of antibodies against HTLV shows that the prevalence increases with increasing age and is not different between males and females; this is evidence of an equal efficiency of transmission from men to women and from women to men [6, 28, 56] with the aid of vertical transmission acting by chance to infect both sexes equally. It is worth mentioning that it is not commonly observed in urban areas [42].

The description of HTLV-1 and HTLV-2 soon led to seroepidemiological studies based on the detection of antibodies against HTLV, which is the usual approach to determine the initial prevalence rates of HTLV according to geographical locations, age and sex, among other variables. Few studies have been conducted strictly with controlled populations to verify the published information from the 1980s and 1990s. Consequently, there have been few attempts to establish the trends in prevalence and incidence rates and the spread of HTLV in Brazil and, particularly in the Amazon region of the country.

## Detection of HTLV in the Amazon region of Brazil

HTLV-1 was described in 1980, and HTLV-2 was described in 1982 [59–62]. Soon, knowledge of their geographical distribution was expanded through the production of seroepidemiological data, which clearly defined the low prevalence (up to 1%) but almost universal presence of HTLV-1 among specific populations (in Europe, the Americas, the Caribbean, and sub-Saharan Africa), reaching more than 30% in some areas of southern Japan [63–66].

HTLV-2, however, showed a distribution limited to intravenous drug users in the USA, Europe, Southeast Asia and among American Indians from North America to South America as well as in the Pygmy tribes in Central Africa [6, 47–49, 66–72]. The prevalence rates were generally low (except for the hyperendemicity among some Amazonian Indian communities), and the association with disease was substantial with HTLV-1 and not usually common with HTLV-2 [73]. In Brazil, associated diseases were initially described in different geographical areas and in specific groups, including blood donors and patients with hematological and neurological diseases [74–83].

In the Amazon region of Brazil, HTLV-1 and HTLV-2 infections were primarily described [63, 84–86], and soon their geographical dissemination expanded [6]. In 1998, HTLV-2 was detected for the first time outside native Indian communities, and both viruses were found in blood donors [82] and HIV-1 carriers [50]. The first cases of diseased persons were described with HTLV-1-associated myelopathy/tropical spastic paraparesis (HAM/TSP) among persons residing on Marajo Island [83], and the presence of both viruses on the east coast of the island was also identified on Afro-descendants [87]. HTLV-1 was described among sex workers, and for the first time, there was a clear geographical link when the virus was identified among Japanese immigrants in the Amazon originating from Kyushu, a highly endemic area of HTLV-1 in Japan [88].

HTLV human infections in the Amazon region of Brazil have been recorded by several studies that considered widely different populations, including blood donors, pregnant women, urban familial aggregates and native indigenous people. These investigations comprise the epidemiological picture in the North region of the country and will be presented and discussed further in the following sections.

## The Amazon region of Brazil

The Amazon is a large geographical area involving six countries, and Brazil holds the largest area in the system. The Amazon region of Brazil (ARB) involves nine federative States and an area of 5.1 million km^2^, which represents 60% of the country but is inhabited by approximately 15% of the Brazilian population.

There is a large difference in demographic, social, cultural and development between the ARB and the rest of the country, which is evidenced in some commonly used markers related to the health and education of the population and its level of welfare and development. Historically, the ARB was always left behind by policies given that the population of the region represents only 13% of the gross internal revenue of the country, has a higher rate of illiteracy (12.9% vs. 10.2%), a slightly higher infant mortality rate (18.6 vs. 15.9 per 1000) and a lower life expectancy (72 vs. 76 years) in comparison to the other areas of Brazil [89].

On the other hand, the ARB is a unique geographical area, considering the diversity of humans and other living species. Approximately half of the so far undescribed living organisms on earth (plants, vertebrates, and microorganisms, among others) reside within the ARB [90].

The history of HTLV is a fascinating one regarding its possible origin in the African continent and its spread in different directions according to human migration routes [4], and the ARB is of paramount importance because of the presence of a specific strain of the virus that originated during human migration into the area and its further dissemination to other geographical areas in the country and abroad [36, 51, 91–97].

## Epidemiological data of HTLV-1 and HTLV-2 in the Amazon region of Brazil

**Fig. 1:**
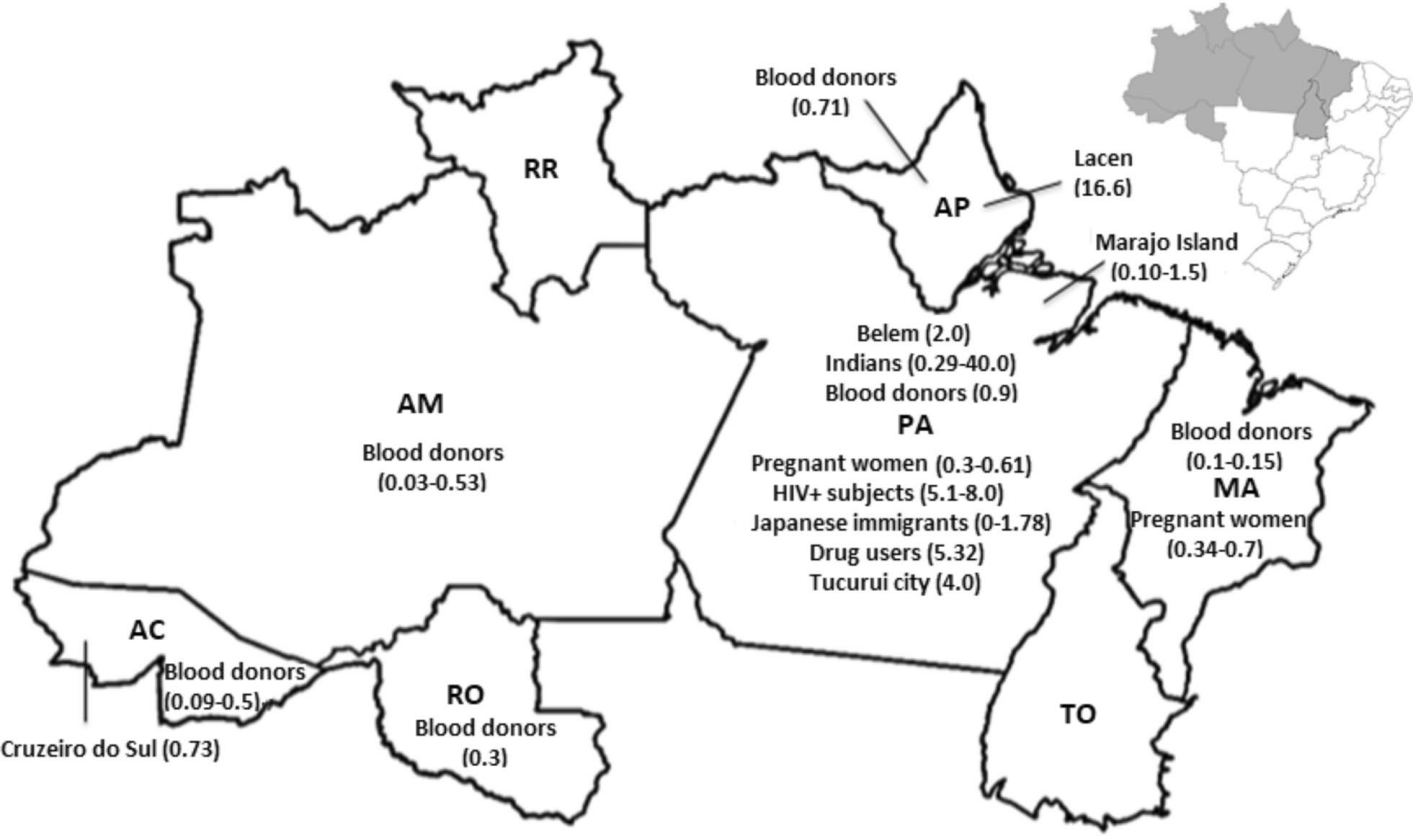
Representation of the Amazon region of Brazil with the results of prevalence rates of HTLV-1/2 infection in population groups

Human infections by HTLV in the Amazon region of Brazil have been recorded by several studies involving blood donors [82, 98–102], pregnant women [103–106], urban familial aggregates [42] and native Indians [6, 56] (Fig. 1).

The isolation of HTLV-1 and HTLV-2 led to the manufacturing of serological assays to detect human antibodies against the viruses, and several studies were immediately performed describing prevalence rates around the world. South American countries reported quite different figures with large variations, including in Argentina (0.07%), Chile (0.73%), Venezuela (6.8%), Colombia, where the prevalence was higher in low geographical areas (4.3%) than in higher areas (0.73%) of the country, and in French Guiana (6.7–13.1%) [107–110]. It is important to bear in mind that these figures are rarely comparable as there are no uniformity of the different population groups investigated (age, sex, sampling, laboratory assays, among others). It is relevant to mention that the same variables are the cause to find such conflicting figures also described in Brazil where the mean prevalence was initially thought to be approximately 0.41%, but there was a large range of prevalence rates from 0.08% in Manaus and Florianopolis to 1.35% in Salvador [98]. It was common to use serological assays prepared with different strains of HTLV and different targets of the reagents to detect the antibodies, and this technique is one of the possible reasons for such a variation in the figures found.

In a second round of prevalence studies, a more comprehensive investigation was conducted with national public blood banks [100], and the figures were found to be clearly different. There was a range from 0.4/1000 in the State of Santa Catarina (southern part of the country) to 10/1000 in the State of Maranhao (in the northeast). In the Amazon region of Brazil, the prevalence ranged from 1/1000 in the State of Rondonia to 9.1/1000 in the State of Para [100]. For the purpose of the geopolitical division of the country and economic development, the so-called Amazonia Legal includes three other states: Maranhao, Tocantins and Mato Grosso. Some of the present information and discussion will also include the State of Maranhao.

The main issue of concern presented is the large variation obtained by the different investigations conducted in the same geographical area. The data include the states in the North region of the country, but the results are not different from those in the rest of the country. The discrepancy is shown in Table 1, which summarizes the figures obtained in the different studies.

**Table 1:** Prevalence rates of HTLV-1/2 infection in population groups of the Amazon region of Brazil

Location	Group	Sample size	Prevalence rate HTLV (%)	HTLV-1 (n)	HTLV-2 (n)	HTLV (n)^a^	Reference (#)
Rondonia	Blood donors	–	0.3^a^	–	–	–	[100]
Acre	Blood donors	11,121	0.09	9	3	–	[99]
	Blood donors	–	0.5^a^	–	–	5	[100]
	Blood donors	219	0.46	1	0	–	[101]
	Cruzeiro do Sul	136	0.73	1	0	–	[111]
Amazonas	Blood donors	1200	0.08	1	–	–	[98]
	Blood donors	–	0.53^a^	–	–	–	[100]
	Blood donors	6865	0.14	–	–	–	[112]
	Pregnant women	674	0	–	–	–	[105]
	Skin diseases	1091	0	–	–	–	[112]
	Blood donors	87,402	0.03	16	5	3	[113]
Amapa	Blood donors	–	0.71^a^	–	–	–	[100]
	Afrodescendants	186	0	–	–	–	[114]
	HIV-1	52	0	–	–	–	[118]
	HIV-1	140	0	–	–	–	[114]
	Lacen	30	16,6	5	–	–	[114]
Maranhao	Blood donors	–	0.1^a^	–	–	–	[100]
	Pregnant women	2044	0.34	4	3	–	[104]
	Pregnant women	324	0.7	5			[115]
	Blood donors	365,564	0.15^a^	–	–	72^a^	[102]
Para	Indians	137	31.4^a^	43	–	–	[84]
	Indians	209	10.5	22	–	–	[85]
	Blood donors	–	0.9^a^	–	–	–	[100]
	Indians	314	7.6	1	18	5	[63]
	Indians	1324	0–40	5	104	–	[6]
	Indians	177	5.1	–	9	–	[51]
	Indians	263	0.29	–	77	–	[56]
	Pregnant women	13,382	0.3	39	1	–	[103]
	Pregnant women	324	0.61	2	0	–	[106]
	Tucurui	657	4.7^a^	–	–	–	[116]
	Belem	1059	2	15	5	–	[117]
	HIV-1	149	8.0	4	7	1	[50]
	HIV-1	117	5.1	2	4	–	[118]
	Japaneses	44	0	–	–	–	[85]
	Japanese	168	1.78	3	–	–	[88]
	Afrodescendants (Marajo Island)	259	1.5	3	1	–	[87]
	Marajo Island	1899	0.10	2	–	–	[94]
	Drug users	826	5.32	25	19	–	[55]

^a^Tested only by enzyme immune assay (ELISA)

In Rio Branco (Acre), the investigation of HTLV among blood donors showed that using two enzyme immunoassays, the results were significantly different (0.66% vs. 0.11% of 11,121 samples); the Western blot results confirmed the presence of antibodies in 8 samples for HTLV-1 and 2 for HTLV-2. PCR results confirmed one HTLV-1 and one HTLV-2 reaction. The study shows the nature of conflicting results when comparing those with later results: 0.09% [99] and 0.5% [100]. Mota-Miranda et al. [101] investigated the molecular epidemiology of HTLV-1 and described a prevalence rate of 0.46% among blood donors in 2004, although the sample was smaller than the first investigation by the local group. The state of Acre is an endemic area for malaria, an additional variable that might add confusion because of the misinterpretation of results owing to cross-reactions against HTLV-1 detected in patients infected with *Plasmodium* sp. [111]. Even though, a prevalence of 0.73% was detected among the general population of Cruzeiro do Sul, where malaria is endemic. There was a strong expectation of high prevalence rates in the State of Acre considering the Indians racial mixture of the general population, but so far, there is no evidence of wide distributions of the two viruses.

In Manaus (Amazonas State), the results are also conflicting. The initial prevalence was set at 0.08% [98] and was later described as 0.53% [100] and 0.14% [112] for HTLV-1/2 among blood donors in urban areas. Two other relevant investigations showed that HTLV infection was not present among pregnant women [105] or patients presenting with skin diseases (including dermatitis), leishmaniasis and leprosy [112]. More recently, data were published related to a retrospective prevalence of antibodies in a large group of blood donors (n = 87,402) who were initially screened from 2001 to 2003; however, only 24 persons were confirmed to be infected by HTLV-1 and HTLV-2, which shows the low prevalence of infection in the city of Manaus [113].

The presence of the virus in different areas and populations in the State of Amapa has been investigated a few times, and low prevalence rates of infection (0.71%) have been found among blood donors [100]. The viruses were not found in HIV-1 infected persons [114, 118] or in a quilombo (Afro-descendants in isolated communities that were originally founded by escaped slaves); however, HTLV-1 was found to be present (5/30) among individuals seeking a serological diagnosis in a public health laboratory [114].

In Sao Luiz, the capital city of the State of Maranhao, the highest prevalence rate (1%) among blood banks in Brazil was identified [100], but the prevalence rate among pregnant women ranged from 0.34% (HTLV-1, 0.19% and HTLV-2, 0.15%) [104] to 0.7% (HTLV-1 only) more recently described [115]. Retrospective information on blood donors showed that only 0.15% among more than 365,000 persons tested in the period 2003–2009 were positive for HTLV-1/2 [102], however only 53 persons confirmed the seroreactivity. The frequencies of the identification of both viruses are not significantly different, but again the general prevalence showed figures that are somewhat lower than what was usually expected and previously described in the State of Maranhao.

The State of Para has been the site of the majority of epidemiological studies intending to define prevalence rates of antibodies against HTLV-1/2 with a variety of different populations, and several conflicting results have been obtained. The initial figures indicated that the prevalence rates measured by immunoenzymatic assays ranged from 3.6% to more than 30% for HTLV-1, mostly among Indians populations [84, 85], that the rate was 0.91% among those undergoing blood donation screening [100]. A large distribution of HTLV-2 was shown among Indians communities not only in the State of Para but also in the Amazon area of Brazil, reaching confirmed prevalence rates of more than 40% [6, 51, 56, 63] among some Indian communities and the detection of a new molecular subtype (HTLV-2c) that was soon also described in urban areas outside of the ARB [6, 33, 50, 51, 116]. Prevalence studies also provide interesting information, such as the description of HTLV-2b among blood donors in Belém, which stresses the need for ongoing molecular epidemiology investigations [116]. A comprehensive investigation detected HTLV-1 among 0.3% (n = 39) of 13,382 pregnant women in Belém and one person infected with HTLV-2 [103]. Later, another prevalence rate of 0.61% was detected in pregnant women in Belém [106]. A prevalence rate (4.7%) of antibodies against HTLV, detected by enzyme immune assay, was described among residents along the shores of the Tucurui hydroelectric power plant [117]. This was a rather high figure for urban communities, however there was no confirmation of reactivity by other laboratory methods. The most recent investigation in Belém considered the prevalence of antibodies against HTLV among 1059 inhabitants and showed a 2% positivity for antibodies against both HTLV-1 (n = 15) and HTLV-2 (n = 5) [118].

Two different studies dealt with HTLV/HIV-1 co-infections. The first approach found a prevalence rate of 8% (12/149) for individuals, mostly males (n = 10), positive for HTLV-1 (n = 4) and HTLV-2 (n = 7) [50]. A later approach detected a prevalence rate of 5.1% (6/117) for co-infections [119] with HTLV-1 (n = 02) and HTLV-2 (n = 04), and those individuals were mostly female (n = 4). This clearly indicated the change in sex predominance in the HIV-1 epidemic in the city of Belém.

The initial approach of Japanese immigrants did not result in HTLV reactivity [85], but later, the prevalence of 1.78% of HTLV-1 was found in immigrants from Kyushu residing in the ARB [88]. The Marajo Archipelago also showed different figures according to the population group investigated. It was higher in an epidemiologically semi-closed quilombo than the average of four municipalities (1.5% vs. 0.1%, respectively) investigated [87, 94]. Since the initial detection of HAM/TSP in patients from the Marajo Archipelago, in the North region of Brazil [83], HTLV-1-infected male and female patients with different signs of clinical severity of neurological disease have been described [20]. Although there have been continuous descriptions of persons with other diseases, including dermatological symptoms [120], no hematological disorders, so far, have been associated with HTLV-1 infection in the ARB.

Fairly rapid changes in the epidemiology of HTLV-1/2 are occurring in the ARB. A recent and extensive study was performed in the State of Para, which detected an intermediate prevalence of 4.3% with almost the same frequencies of HTLV-1 (n = 25) and HTLV-2 (n = 19) among drug users [55]. Both viruses are widely distributed in the Amazon region of Brazil and particularly among this population, who especially need close attention in terms of the control and prevention of infection as they are key participants in the spread of the virus.

## Conclusions

Epidemiological studies in the ARB, as in other areas of the country and abroad, have been plagued by imprecise sampling (“grab samples” was common), which generated inadequate and conflicting results among several studies. Groups of sub-populations were consistently chosen, with the equivocal assumption of a general figure for most of the region, leading to a selection bias that rendered untrustworthy results. The selection of blood donors, the selection of diseased population subgroups, and the retrospective collection of data are some of the examples that should be avoided in future prevalence studies. Relevant population groups should be selected and preferably using multiple centers of study working together to increase sample size. It is relevant to stress that confirmatory tests should be always used to make sure the information is not equivocal and the absence of correct information should be put ahead to avoid misinterpretations. Urban populations should be the optimal target to answer important questions such as the following: who is mostly affected by this neglected infection? Who should receive future preventive measures such as vaccines? Which risk factors are relevant for the transmission of the virus?

HTLV-1 is an important human pathogen and the only human retrovirus associated with a large array of diseases, including lymphoma/leukemia. However, in the possible event of the development of a vaccine in the near future, health authorities will not be able to immediately define the population at risk who should receive it, either in the ARB or in other areas of the country. New and authoritative epidemiological information should be gathered to gauge the actual need for such a product. Australian aboriginal people are examples of persons at high risk for morbidity and mortality associated with the wide dissemination and high prevalence of HTLV-1. Respiratory diseases recently also described in the ARB [121] are new and dangerous facets of the infection that can truncate productive lives, which is a compelling reason to revitalize HTLV-1 epidemiological studies in the ARB, elsewhere in the country and worldwide.

Small epidemiologically closed or semi-closed human aggregates are easier with regard to the implementation of preventive measures, although transmission in such communities is further enhanced by the mother to child transmission route in utero, during birth and during breast feeding both perinatal and after birth, which corroborates the formation of familial aggregates. The most recent report from our laboratory has shown that Asurini and Arawete Indian tribes have remained free from HTLV-1/2 infections due to cultural and social isolation from the infected neighboring tribes and villages [122]. Indian communities from the North region of Brazil usually experience hyperendemic infections with HTLV-2, which is less pathogenic than HTLV-1; this is a more favorable situation compared to what is found among native aboriginal people in Australia, where hyperendemicity exists with the more pathogenic HTLV-1 [30, 123].

It is reasonable to infer that the general prevalence of the virus should be decreasing because of the general policies regarding testing blood donations and some efforts to disseminate general knowledge of the virus, but so far this has not been substantiated, as conflicting prevalence results generate confusion regarding the adequate delivery of information. National associations of HTLV-infected persons are active and give full support to the elimination of the virus, but their messages are not always disseminated through the appropriate channels. Although the existing preventive campaigns are not aggressive, they should receive full official support to attain success in the future, particularly as proposals are being made with regard to virus eradication [124].

## Data Availability

Not applicable.

## Abbreviations

HTLV: Human T-lymphotropic virus
HAM/TSP: HTLV-1 associated myelopathy/tropical spastic paraparesis
ARB: Amazon region of Brazil
PCR: Polymerase chain reaction
